# Measurement of the sound velocity of shock compressed water

**DOI:** 10.1038/s41598-021-84978-0

**Published:** 2021-03-17

**Authors:** Hua Shu, Jiangtao Li, Yucheng Tu, Junjian Ye, Junyue Wang, Qili Zhang, Huiru Tian, Guo Jia, Zhiyu He, Fan Zhang, Zhiyong Xie, Xiuguang Huang, Wenbin Pei, Sizu Fu

**Affiliations:** 1 Shanghai Institute of Laser Plasma, China Academy of Engineering Physics, ShangHai, 201102 China; 2 Fluid Institute of Fluid Physics, China Academy of Engineering Physics, Mianyang, 621900 Sichuan China; 3 State Key Laboratory for Environment-Friendly Energy Materials, Southwest University of Science and Technology, Mianyang, 621010 Sichuan China; 4 Center for High Pressure Science and Technology Advance Research, ShangHai, 201111 China; 5 Institute of Applied Physics and Computational Mathematics, PeiJing, 102311 China

**Keywords:** Giant planets, Phase transitions and critical phenomena

## Abstract

The sound velocities of water in the Hugoniot states are investigated by laser shock compression of precompressed water in a diamond anvil cell. The obtained sound velocities in the off-Hugoniot region of liquid water at precompressed conditions are used to test the predictions of quantum molecular dynamics (QMD) simulations and the SESAME equation-of-state (EOS) library. It is found that the prediction of QMD simulations agrees with the experimental data while the prediction of SESAME EOS library underestimates the sound velocities probably due to its improper accounting for the ionization processes.

## Introduction

Water at extreme conditions is one of the most concerned topics due to its significant abundance in icy giant planets like Uranus and Neptune^1–5^, as well as extrasolar ‘hot Neptunes’ and ‘mini-Neptunes’^6,7^. The thermodynamic states of water inside the icy giant planets can be approximated by isentropic lines^8^, along which the derivative of pressure with respect to density is the sound speed^9^. Since the sound speed is usually measured on a Hugoniot line, the information of the intersection of the Hugoniot line with the local isentropic line is intrinsically carried by the sound speed. Therefore, sound speed information is important if a planetary interior model needs to be built purely based on experimental equation of state data. Apart from this, the sound speed data is also valuable to test equation-of-state (EOS) models since as a second derivative of the Gibbs free energy they are generally more sensitive to the minor differences between various EOS models.

The report on the sound speed of water at high pressure is scarce. However, the method of measuring the sound speed of a material has been well established, especially in the case of gas gun experiment^10^, where the thicknesses of the flyer and target can be measured precisely for determining the speed of the overtaking rarefaction waves. The technique becomes difficult to apply in the case of laser shock compression, which aims at reaching higher pressure along the Hugoniot line. The difficulty is related to the fact that the position of the ablation front is changing with respect to the laser intensity, the target material and the amount of ablated material^11^. Recently, a new method is demonstrated to determine the sound speed of quartz under laser shock compression, which uses the bending boundary formed by the propagation of the lateral rarefaction wave into the shock compressed region, as probed by a line-imaging Velocity Interferometer System for Any Reflector (VISAR)^12^. In addition to that, there are other indirect methods proposed to determine the sound speed of materials under laser shock compression, such as the detection of acoustic perturbations in the target with reference to that in a standard material^13–15^. The establishment of these methods makes it possible to determine the sound speed of water under laser shock compression.

In this work, we report the measurement of sound speed of water in the case of a combination of pre-compression using diamond anvil cell (DAC) and laser shock compression. This technique makes it possible to obtain the sound speed of water in the off-Hugoniot region with reference to the Hugoniot measurement of liquid water. The experimental details and the results will be presented in the Experimental and Result and discussion sections.

## Experiment

**Figure 1:**
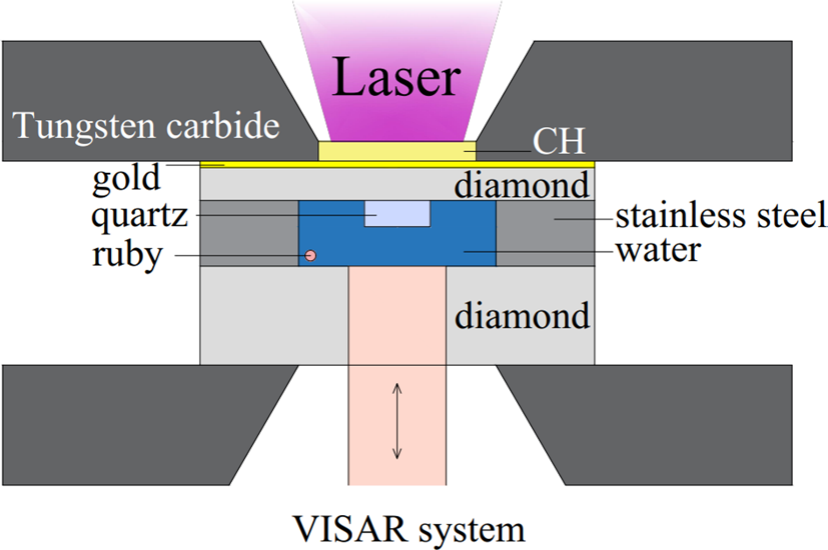
A schematic of experimental setup for laser shock compression of precompressed water in a DAC. For the DAC precompression, the fluorescence peak of ruby is used to infer the initial pressure. For the laser shock compression, a VISAR is used to determine both the Hugoniot state and the sound speed.

Figure 1 shows a schematic of the target assembly for water as well as the diagnostics. Doubly distilled pure water was statically compressed to about 0.57 GPa (about 1.16 g/cm^3^ in density) between two diamond anvils. The front anvil (150 μm in thickness) was coated by a gold film (1.5 μm in thickness) to eliminate preheating effects and a polypropylene (CH) film (25 μm in thickness) to serve as the laser ablator. The rear anvil (1200 μm in thickness) has an anti-reflection coating (with respect to the VISAR probe light at 660 nm) to enhance the signal-to-noise ratio of the VISAR signals. In the chamber between the two anvils, there are a quartz plate, a ruby particle, and the water sample. The quartz plate (with a 200 nm aluminum coating on the side contacting the diamond) serves a standard material for performing impedance matching calculation to determine the Hugoniot state^16^. Under static compression, the fluorescence signal from the ruby particle is used to determine the pressure of water^17–19^. The density of water is inferred according to the well-determined equation of state up to 25 GPa^20^.

For the dynamic compression, a square laser pulse (about 3 ns in duration, 351 nm in wavelength, 800–1500 J in energy) generated from the Shenguang-II laser platform, is focused on the CH film with a spot size of about 0.65 mm in diameter. The interaction between the intense laser pulse and the CH film allows a strong shock wave to propagate towards the statically compressed water sample. As shown in Fig. 1, the shock wave velocity is monitored by a velocity interferometer system for any reflector (VISAR) system. Since both the shock velocities before and after the arrival of the shock wave at the quartz-water interface are determined, the particle velocity of shocked water can be inferred using the impedance matching method^21,22^. As a consequence, both the pressure and density of water after dynamic compression can be determined using the Hugoniot relations.

**Figure 2:**
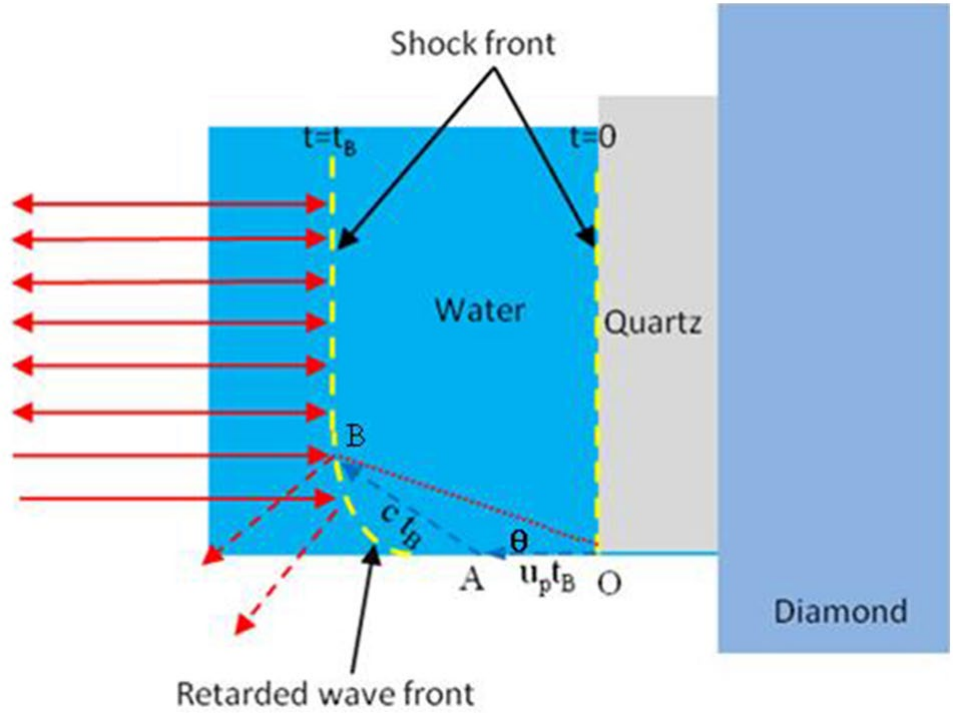
An illustration of how the shock wave front bends due to the propagation of the lateral rarefaction wave.

The sound velocity of water in the Hugoniot state can be determined from the bending boundary of the shock wave front due to the interaction between the lateral rarefaction wave and the shock compressed region^23^, as proposed by Li et al.^24^. This method is briefly illustrated in Fig. 2. Due to the propagation of the lateral rarefaction wave, whose speed is essentially the sound speed in the Hugoniot state, the shock wave front narrows down in the lateral direction. The trace of the bending boundary can be related to the triangle OAB illustrated in Fig. 2 and the sound speed (*c*) is expressed as,

1 $$ c = u_{s} \sqrt {\left( {\frac{{u_{s} - u_{p} }}{{u_{s} }}} \right)^{2} + \tan^{2} \theta } $$

where, *u*_*s*_ is the shock velocity. *u*_*p*_ is the particle velocity. *θ* is the bending angle of the shock wave front which is equivalent to the angle formed by OA and OB in Fig. 2. Therefore, with the shock velocity monitored by the VISAR system and the particle velocity determined by the impedance matching method, the sound speed of water can be obtained from the bending boundary of the shock wave front.

## Results and discussion

**Figure 3:**
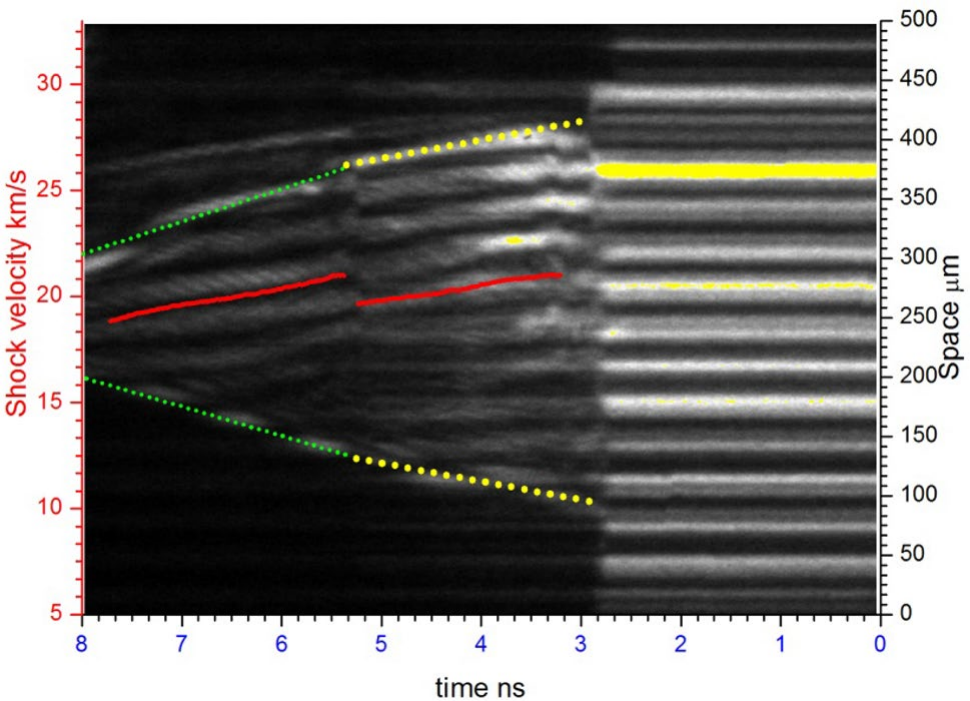
The bending boundary of the shock wave front monitored by the VISAR system. The trace of the bending boundary is highlighted by the dotted line which is yellow in the quartz and green in the water. The shock velocities for impedance matching calculation are also shown by the red solid line.

**Figure 4:**
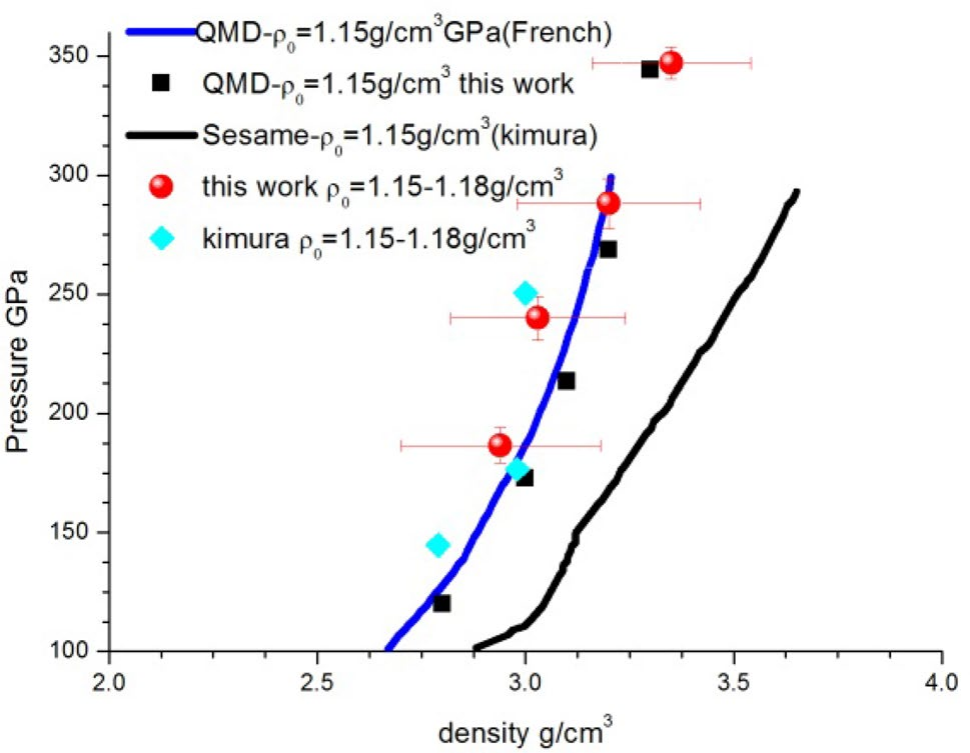
Density and pressure relations for shocked water with initial density of 1.15 g/cm3. Our experiment data in red circle and Kimura^15^ in cyan diamond, QMD EOS model (kimura’s in blue line, this work in black square) and Sesame Model in black line.

The VISAR image of the bending boundary of the shock wave front is shown in Fig. 3. From this VISAR image, we obtained two pieces of information. One is the shock velocities in the quartz and in the water, which can be used to determine the Hugoniot state of water at the quartz-water interface. The other is the associated sound velocity of water at the quartz-water interface, derived from the trace angle as illustrated in Fig. 2. Four shots of water with nearly same initial precompressed pressure(0.6 GPa) were performed. The experimental conditions of initial precompressed water (density) and shocked water (shock velocity, pressure, density and sound velocity) are listed in Table 1. Figure 4 shows the comparison between our data with previous data.

**Table 1:** Experimental conditions of initial precompressed water (density) and shocked water (shock velocity, pressure, density). Uncertainties are given in parentheses.

Shot	ρ_0_ (g/cm^3^)	U_sW_ (km/s)	P_W_ (GPa)	ρ_w_ (g/cm^3^)	C (km/s)
1	1.166	19.72 (0.19)	288.1 (10.7)	3.20 (0.22)	16.12 (1.45)
2	1.171	18.26 (0.18)	240.0 (9.2)	3.03 (0.21)	14.54 (1.20)
3	1.153	16.32 (0.16)	186.6 (7.4)	2.94 (0.24)	14.05 (1.30)
4	1.171	21.20 (0.21)	342.2 (6.8)	3.35 (0.19)	17.70 (1.60)

**Figure 5:**
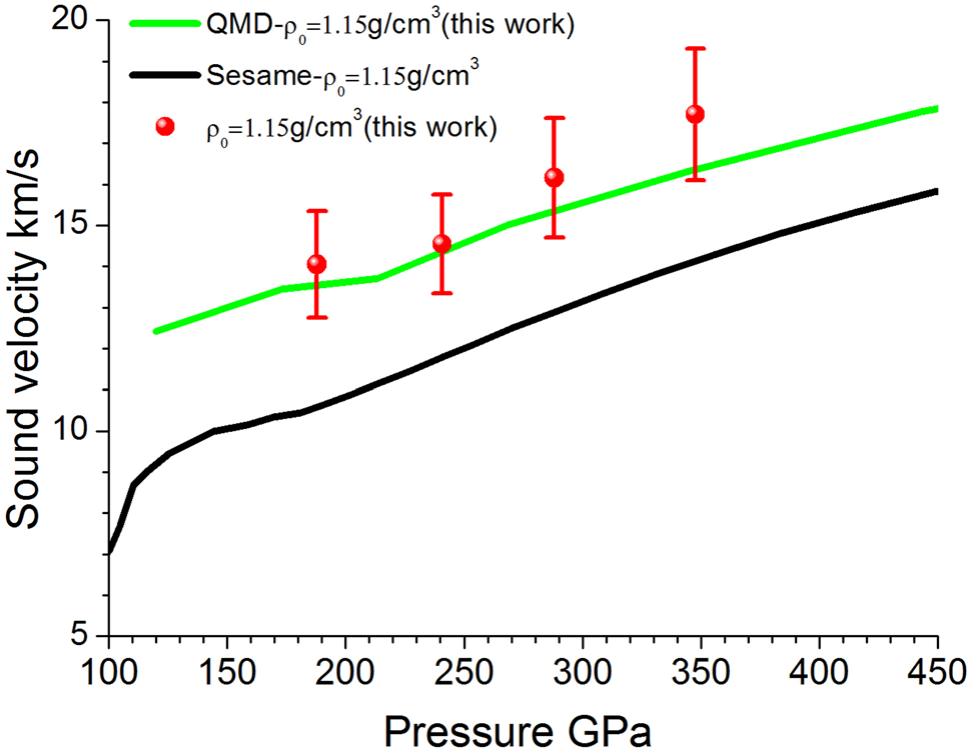
The variation of the sound velocity of water with its Hugoniot pressure: a comparison between experimental data and theoretical predictions.

The variation of the sound velocity of water with respect to the Hugoniot pressure is shown in Fig. 5. It is shown that the sound velocity increases with the Hugoniot pressure, in good agreement with the prediction of quantum molecular dynamics (QMD), which uses Vienna Ab Initio Simulation Package (VASP) to calculate the electronic structure of 54 water molecules in a periodic simulation box. Projector augmented wave (PAW) pseudopotentials with a cutoff energy of 1000 eV for electron–ion interaction and Perdew-Burke-Ernzerhof (PBE) exchange–correlation functional^25–27^ are used. For the molecular dynamics simulation, the time step for ion motion varies from 0.35 to 1.0 fs according to the temperature. The sound velocity is derived from the following relationship:

2 $$ c^{2} = \left( {\frac{\partial P}{{\partial \rho }}} \right)_{T} + \frac{T}{{C_{V} \rho^{2} }}\left( {\frac{\partial P}{{\partial T}}} \right)_{\rho }^{2} $$

Here, the differential terms are determined from the slopes of isothermal or isochoric lines in the pressure-density and pressure–temperature spaces, and the heat capacity at constant volume is determined from the slopes of isochoric lines in the internal energy-temperature space. In contrast with the good agreement between the experimental data and QMD result, the SESAME equation-of-state library^28,29^ predicts a much lower sound velocity along the Hugoniot pressure. This is probably because the ionization processes, which was not well accounted in the model of Ree^29^, plays an important role and number of freedoms released result in the increase of sound velocity.

## Conclusions

The sound velocities of water in the Hugoniot states are determined by monitoring the propagation of lateral rarefaction waves into the shock compressed region using a VISAR system. The Hugoniot states are generated by laser shock compression of precompressed water in a DAC, which lies in the off-Hugoniot region of liquid water at ambient conditions. The relation between the sound velocity and the Hugoniot pressure indicate that QMD is more accurate in predicting the sound velocity of water than the widely used SESAME equation-of-state library, which accounts poorly for the ionization processes.
